# Supplementary material for: Divergence of compost extract and bio-organic manure effects on lucerne plant and soil
Source: PeerJ. 2017 Sep 6;5:e3775. doi: 10.7717/peerj.3775 (PMC5591637; doi:10.7717/peerj.3775)
Supplement: Table S1 — Abbreviations are: DM (dry matter), SB (shoot biomass), RB (root biomass), T B (total biomass), CP (crude protein), NDF (neutral detergent fiber), CDOM (cellulase digestible organic matter), ME (metabolisable energy) , NNI (number of nodules for inoculated species), NNN (number of nodules for non-inoculated species), NWI (nodule weight for inoculated species), NWN (nodule weight for non-inoculated species), SN (stem N), SP (stem P), SK (stem K), LN (leaf N), LP (leaf P), LK (leaf K), SON (soil N), SOP (soil P), SOK (soil K), MBC (microbial biomass Carbon ), MBN (microbial N), MR (microbial respiration rate), QCO2 (metabolic quotient), BD (bulk density), WHC (water holding capacity), OM (organic matter), AN (alkaline N ), AP (available P), AK (available K). (*: 0.01 <P < 0.05, **: 0.001 <P < 0.01, ***: P < 0.001, respectively, and ns indicates not significant, P > 0.05). [file peerj-05-3775-s002.doc]

**Table S1**  *F*-values statistics for the effect of compost extract (CE), bio-organic manure (BOM) and CE +BOM (CEBOM) application on plant species and soil parameters (N=20)

|  | SB  (g DM/pot) | RB  (g DM/pot) | TB  (g DM/pot) | CP  (% DM) | NDF  (% DM) | CDOM  (% DM) | ME  (% DM) | NNI  (#/plant) | NNN  (#/plant) | NWI  (mg) | NWN  (mg) |
| --- | --- | --- | --- | --- | --- | --- | --- | --- | --- | --- | --- |
| CE | 4.0** | 2.9ns | 4.2** | 16.8** | 19.0** | 23.7** | 32.1** | 6.6** | 8.4** | 9.6** | 7.2** |
| BOM | 5.7** | 2.5ns | 4.5** | 22.5** | 2.2* | 5.7* | 9.1** | 8.4** | 6.6** | 9.6** | 6.6** |
| CEBOM | 9.8** | 8.3** | 6.0** | 15.8** | 13.5** | 15.7** | 10.5** | 5.4** | 5.4** | 5.4** | 5.4** |
|  | S_N_  (g/kg) | S_P_  (g/kg) | S_K_  (g/kg) | L_N_  (g/kg) | L_P_  (g/kg) | L_K_  (g/kg) | SO_N_  (g/kg) | SO_P_  (g/kg) | SO_K_  (g/kg) | MBC  (mg/kg) | MBN  (mg/kg) |
| CE | 2.1* | 3.0** | 4.8** | 4.6** | 3.5** | 4.0** | 3.9** | 2.7** | 2.6** | 2.5** | 1.2* |
| BOM | 5.1** | 3.1** | 3.2** | 1.6* | 3.0** | 4.2** | 2.6** | 2.9** | 2.2** | 2.0* | 0.1ns |
| CEBOM | 5.2** | 4.6** | 3.5** | 4.6** | 7.3** | 9.2** | 4.6** | 2.3** | 3.1** | 3.1** | 3.1** |
|  | MR  (mg/kg/d) | QCO_2_  (mg C/kg) | Bacteria  (×10^3^cfu /g) | Fungi  (×10^3^cfu /g) | pH | BD  (g/cm^3^) | WHC  (%) | OM  (g/kg) | AN  (mg/kg) | AP  (mg/kg) | AK  (mg/kg) |
| CE | 4.1* | 1.9* | 1.9* | 3.3** | 1.1ns | 2.5* | 2.2ns | 5.8** | 6.2** | 6.1** | 7.8** |
| BOM | 13.7** | 4.1* | 12.3** | 1.2* | 1.4ns | 3.5* | 12.5* | 6.5** | 9.0** | 8.4** | 11.7** |
| CEBOM | 11.1** | 10.1** | 9.1** | 10.0** | 1.8ns | 8.8** | 13.5* | 5.7** | 8.2** | 6.2** | 8.2** |

Abbreviations are: DM (dry matter), SB (shoot biomass), RB (root biomass), TB (total biomass), CP (crude protein), NDF (neutral detergent fiber), CDOM (cellulase digestible organic matter), ME (metabolisable energy), NNI (number of nodules for inoculated species), NNN (number of nodules for non-inoculated species), NWI (nodule weight for inoculated species), NWN (nodule weight for non-inoculated species), S_N_ (stem N), S_P_ (stem P), S_K_ (stem K), L_N_ (leaf N), L_P_ (leaf P), L_K_ (leaf K), SO_N_ (soil N), SO_P_ (soil P), SO_K_ (soil K), MBC (microbial biomass Carbon), MBN (microbial N), MR (microbial respiration rate), QCO_2_ (metabolic quotient), BD (bulk density), WHC (water holding capacity), OM (organic matter), AN (alkaline N), AP (available P), AK (available K).

(*: 0.01< *P* < 0.05, **: 0.001< *P* < 0.01, ***: *P* < 0.001, respectively, and ns indicates not significant, *P* > 0.05).
