# Supplementary material for: Divergence of compost extract and bio-organic manure effects on lucerne plant and soil
Source: PeerJ. 2017 Sep 6;5:e3775. doi: 10.7717/peerj.3775 (PMC5591637; doi:10.7717/peerj.3775)
Supplement: Table S2 — Plants were harvested 13 weeks after planting (n = 20 pots, 4 plants per pot). Different letters within a column indicate significant differences (P < 0.05) between treatments. LSD multiple comparisons were used. [file peerj-05-3775-s003.docx]

**Table S2** Effects of compost extract (CE), bio-organic manure (BOM) and CE + BOM (CEBOM) application on the nodulation of lucerne plants

| Treatment | Number of nodules (no./plant) | | |  | Total nodule weight (mg) | | |
| --- | --- | --- | --- | --- | --- | --- | --- |
|  | Inoculated | Non-inoculated | average |  | Inoculated | Non-inoculated | average |
| Control | 20.5+5.2b | 9.0+1.9c | 14.8+2.1c |  | 2.41+0.6b | 0.99+0.0b | 1.70+0.8b |
| CE | 29.7+5.5a | 19.2+2.2a | 24.5+4.3a |  | 5.13+1.0a | 2.35+0.3a | 3.74+2.0a |
| BOM | 11.9+3.1c | 12.2+0.4c | 12.1+2.1d |  | 1.56+0.3c | 1.19+0.1b | 1.38+0.6c |
| CEBOM | 20.1+5.2b | 16.1+2.8b | 18.1+1.6b |  | 2.22+0.4b | 1.43+0.1b | 1.83+0.1b |

Plants were harvested 13 weeks after planting (n=20 pots, 4 plants per pot).

Different letters within a column indicate significant differences (*P* < 0.05) between treatments. LSD multiple comparisons were used.
