# Supplementary material for: Divergence of compost extract and bio-organic manure effects on lucerne plant and soil
Source: PeerJ. 2017 Sep 6;5:e3775. doi: 10.7717/peerj.3775 (PMC5591637; doi:10.7717/peerj.3775)
Supplement: Table S3 — Different letters within a column indicate significant differences (P < 0.05) between treatments. LSD multiple comparisons were used. [file peerj-05-3775-s004.docx]

**Table S3** Effect of compost extract (CE), bio-organic manure (BOM) and CE + BOM (CEBOM) application on soil N, plant N, alkaline N and microbial biomass N (MBN) inoculated with lucerne rhizobium strains

| Treatment | Stem N  (g/kg) | Leaf N  (g/kg) | Soil N  (g/kg) | Alkaline N  (mg/kg) | MBN  (mg/kg) |
| --- | --- | --- | --- | --- | --- |
| Control | 2.21b | 3.32c | 0.95b | 65.48c | 62.80d |
| CE | 2.88a | 5.09a | 1.49a | 128.67a | 81.00a |
| BOM | 2.33b | 4.01b | 1.00b | 96.48b | 70.10c |
| CEBOM | 2.72a | 4.89a | 1.35a | 99.35b | 76.48b |

Different letters within a column indicate significant differences (*P* < 0.05) between treatments. LSD multiple comparisons were used.
